# Avulsion fracture of the calcaneus treated with a knotless suture bridge and cannulated cancellous screw: a case report

**DOI:** 10.1093/jscr/rjag810

**Published:** 2026-09-11

**Authors:** Yusuke Yoshimoto, Tadaki Okayama, Issei Onari, Shingo Takagawa

**Affiliations:** Department of Orthopaedic Surgery, Noto General Hospital, 6-4 Fujihashi-machi, Nanao, Ishikawa 926-0816, Japan; Department of Orthopaedic Surgery, Noto General Hospital, 6-4 Fujihashi-machi, Nanao, Ishikawa 926-0816, Japan; Department of Orthopaedic Surgery, Noto General Hospital, 6-4 Fujihashi-machi, Nanao, Ishikawa 926-0816, Japan; Department of Orthopaedic Surgery, Noto General Hospital, 6-4 Fujihashi-machi, Nanao, Ishikawa 926-0816, Japan

**Keywords:** calcaneal tuberosity avulsion fracture, knotless suture bridge, SpeedBridge, cannulated cancellous screw, osteoporosis, dual fixation

## Abstract

Calcaneal tuberosity avulsion fractures are rare injuries predominantly occurring in elderly women with osteoporosis. Although lag screw fixation has been widely employed, fixation failure remains a significant concern in osteoporotic bone, and no consensus on the optimal fixation method has been established. A 69-year-old woman sustained a right heel injury. Radiographs and computed tomography revealed a Beavis type I calcaneal tuberosity avulsion fracture. Osteosynthesis was performed using cannulated cancellous screw fixation combined with a knotless suture bridge (SpeedBridge). Weight-bearing was initiated at three weeks with an Achilles tendon boot, and the brace was removed at seven weeks. In Beavis type I fractures, reliable fixation with screws alone is difficult. The knotless suture bridge provides vertical compression force for fragment stabilization while eliminating knot prominence, potentially reducing soft-tissue complications. This dual fixation technique may represent a useful option for these fractures in osteoporotic patients.

## Introduction

Calcaneal tuberosity avulsion fractures are relatively rare injuries, accounting for 1% to 3% of all calcaneal fractures [1]. Unlike intra-articular calcaneal fractures caused by axial loading, these are extra-articular injuries caused by intrinsic forces from the powerful contraction of the gastrocnemius-soleus complex transmitted through the Achilles tendon, combined with extrinsic forces from low-energy trauma involving forced dorsiflexion of the ankle [2]. These fractures predominantly occur in elderly women with poor bone quality associated with osteoporosis and diabetes mellitus, and the fragile bone fragments are prone to re-displacement after fixation [2, 3]. Beavis *et al*. [4] classified these fractures into three types: type I (sleeve fracture), type II (beak fracture), and type III (infrabursal avulsion). The displaced fragment, retracted proximally by the Achilles tendon, compresses the thin posterior heel skin, posing a risk of skin necrosis and necessitating semi-urgent surgical intervention [5]. Lag screw fixation has been the most widely employed method; however, screw pull-out and fixation failure remain significant concerns in osteoporotic bone [5, 6]. To overcome these limitations, various alternative techniques have been proposed [5–8]. However, a systematic review reported an overall complication rate of 34.1%, and no consensus on the optimal fixation method has been established [5]. We herein report a case of calcaneal tuberosity avulsion fracture treated with dual fixation using a knotless suture bridge (SpeedBridge, Arthrex) combined with cannulated cancellous screw (CCS), and describe the short-term clinical results.

## Case report

**Figure 1:**
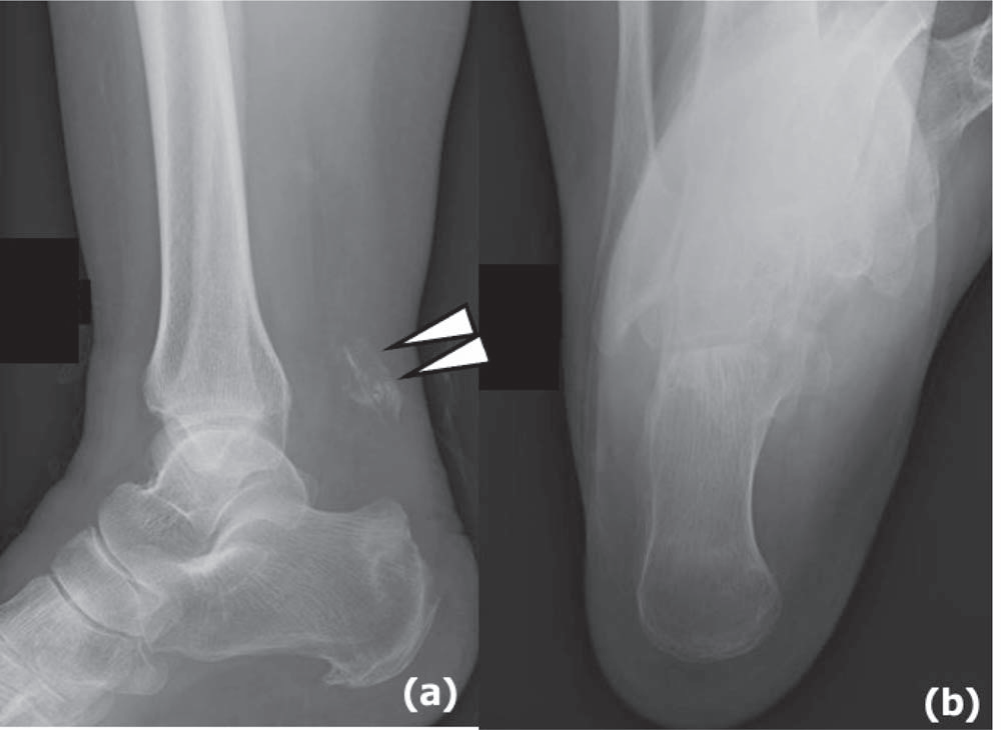
Preoperative radiograph. The sagittal radiograph of the right ankle showing a small avulsion fragment at the posterior calcaneal tuberosity. (a) Sagittal image, (b) axial image.

**Figure 2:**
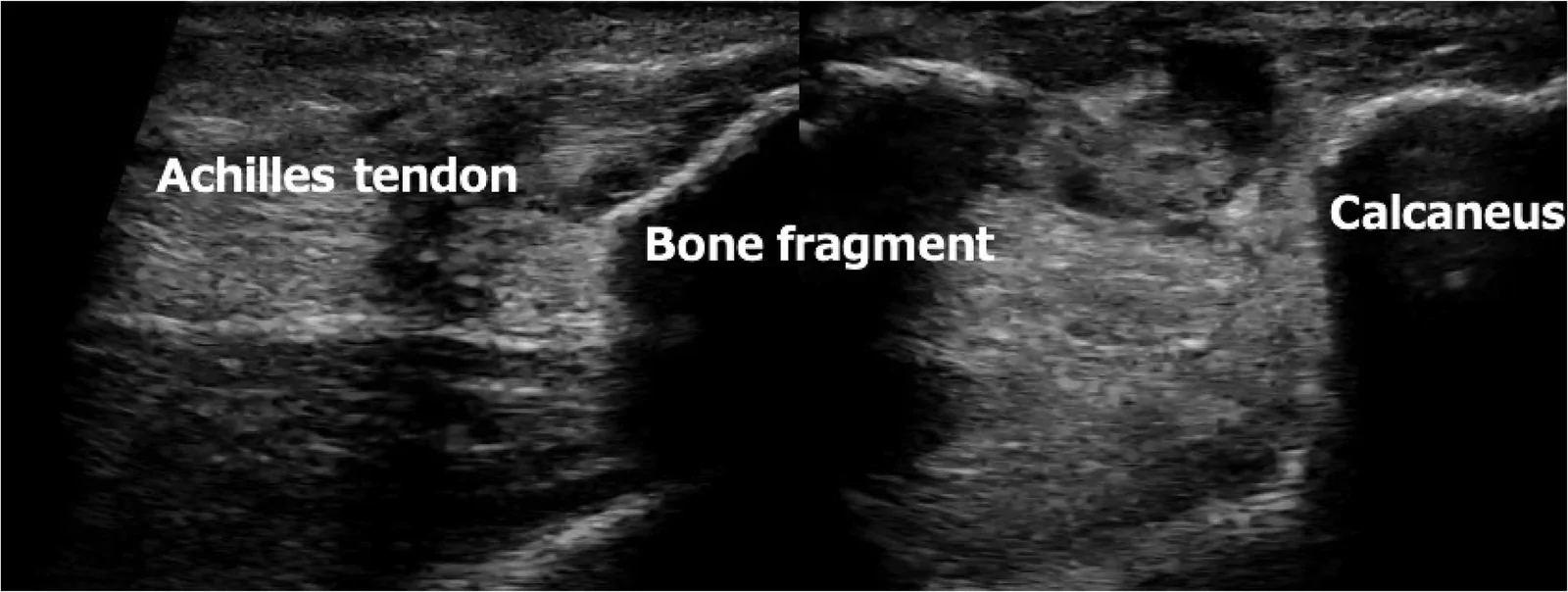
Preoperative ultrasonography demonstrating the Achilles tendon attached to the avulsed bone fragment with discontinuity from the calcaneal body.

**Figure 3:**
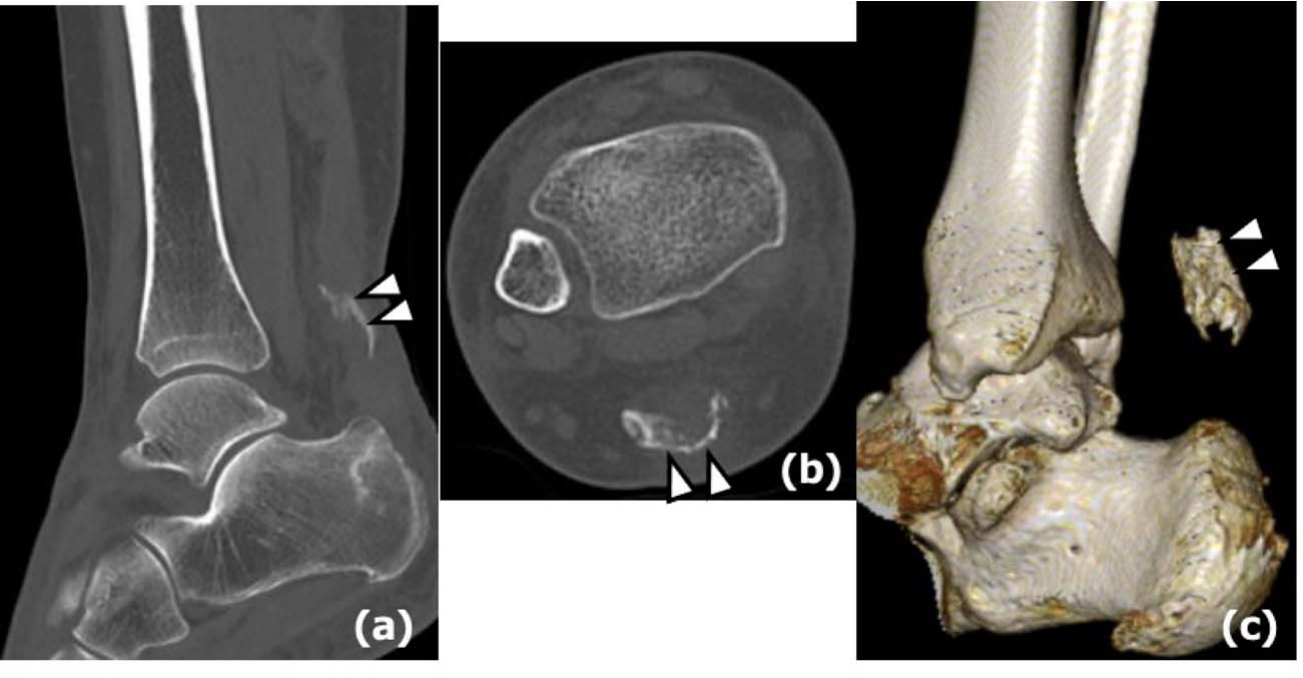
Preoperative CT, showing detailed fracture configuration and fragment size. (a) Sagittal image, (b) axial image, (c) 3D reconstruction.

**Figure 4:**
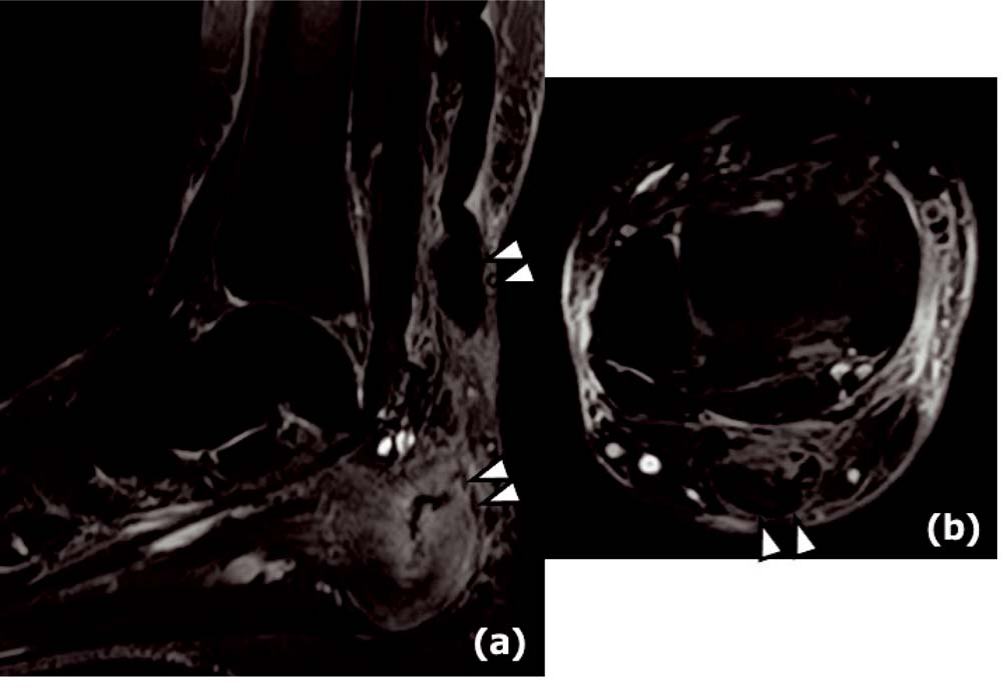
Preoperative MRI (T2 fat-suppressed, sagittal view) showing bone marrow edema of the calcaneus. (a) Sagittal image, (b) axial image.

A 69-year-old woman presented with right heel pain after stepping off a concrete block while cleaning at an elevated position. She visited a local clinic 3 days after injury, where radiographs revealed an avulsion fracture of the calcaneal tuberosity, and was referred to our department on Day 4. On examination, the patient was ambulatory with ankle swelling but without blister formation. Active toe movement was preserved. The Thompson test was positive. Radiographs showed a small bone fragment posterior to the ankle joint (Fig. 1). Based on the Beavis classification, the fracture was classified as type I. Ultrasonography confirmed that the Achilles tendon was attached to a small bone fragment with discontinuity between the fragment and the calcaneal body (Fig. 2). Computed tomography (CT) provided detailed visualization of the fracture configuration and fragment size (Fig. 3). Magnetic resonance imaging (MRI) T2 fat-suppressed images showed bone marrow edema of the calcaneus (Fig. 4).

**Figure 5:**
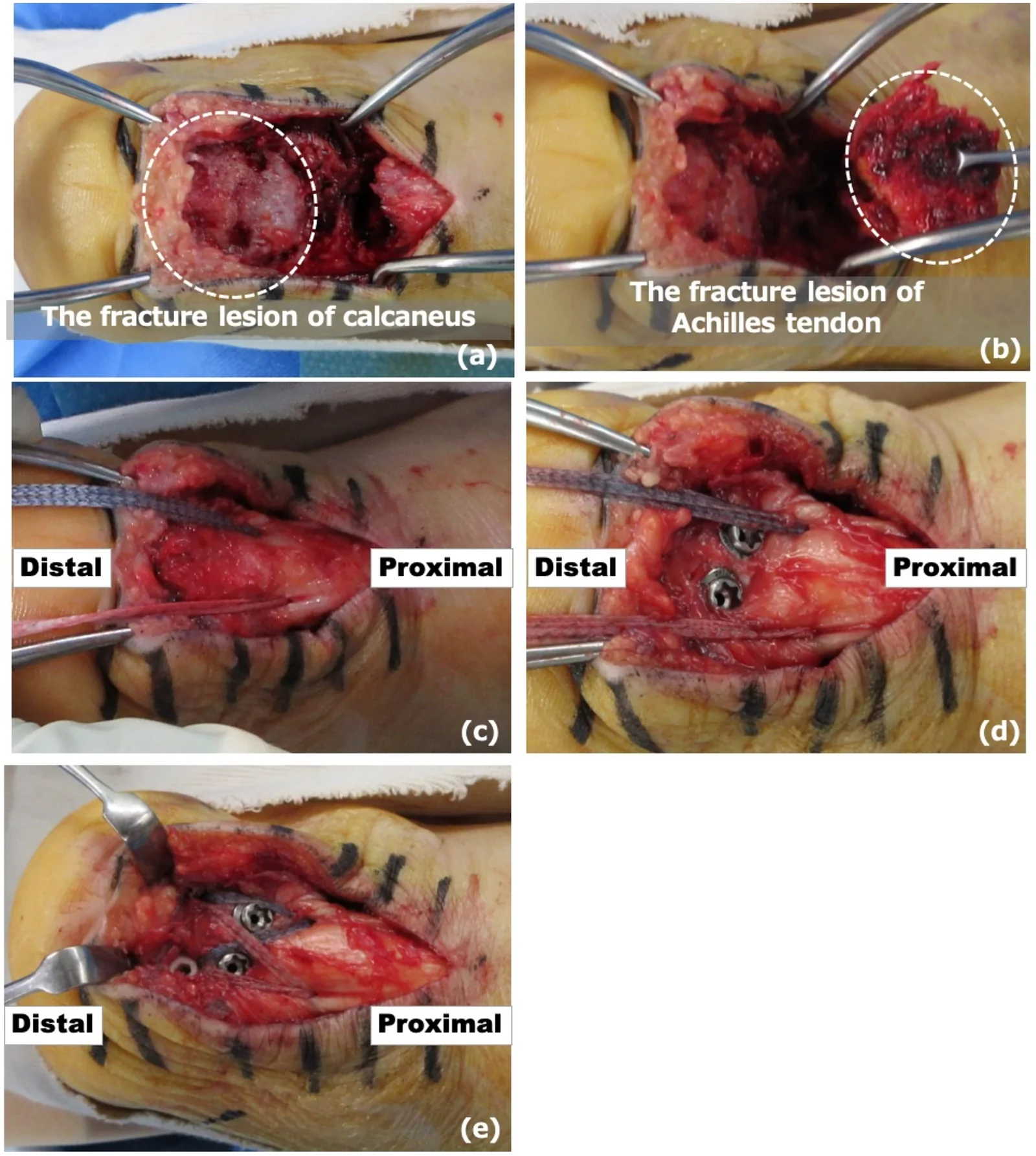
Intraoperative photos. (a) The fracture lesion of calcaneus. (b) The fracture lesion of Achilles tendon. (c) After two soft anchors were driven into the calcaneus and the suture tape was passed through the Achilles tendon. (d) After two 4.0 mm CCS were inserted from the posterior aspect. (e) After forming the SpeedBridge.

**Figure 6:**
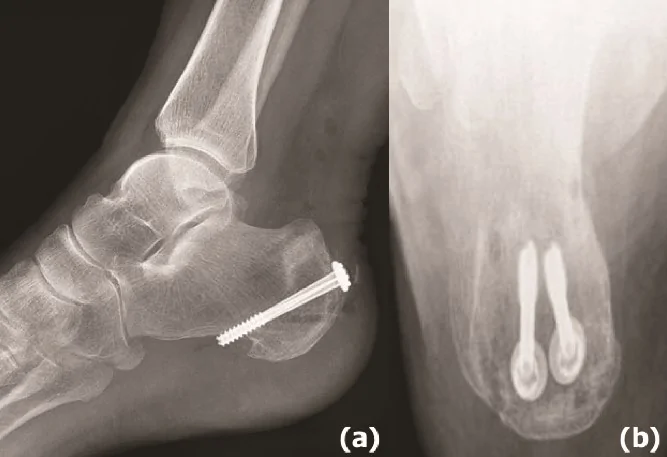
Postoperative radiographs. (a) Sagittal image, (b) axial image.

A 5 cm longitudinal incision was made over the Achilles tendon. The fracture site was refreshed by curettage (Fig. 5a). The small bone fragment attached to the Achilles tendon was retracted using tendon forceps (Fig. 5b). Two soft anchors (BC SwiveLock C 4.75 mm SutureButton Tape Loop, Arthrex) were inserted into the calcaneus, 5 mm proximal to the fracture site, spaced ~2 cm apart (Fig. 5c). The suture tape was passed through the Achilles tendon. While maintaining reduction, two 4.0-mm CCS were inserted from the posterior aspect to apply compression across the fracture site (Fig. 5d). Two additional soft anchors (BC SwiveLock C 4.75 mm, Arthrex) were then inserted into bone holes created 5 mm distal to the fracture line, spaced 2 cm apart. One limb of the suture tape was passed from each side, completing the SpeedBridge construct (Fig. 5e). Postoperative radiographs confirmed satisfactory fixation (Fig. 6).

Immediately after surgery, the foot was immobilized in maximum plantarflexion with a cast, and the patient was kept non-weight-bearing. At three weeks postoperatively, the patient was permitted to bear weight in a heel-raised Achilles tendon boot. Range-of-motion

exercises were also initiated. The heel raise was gradually reduced on a weekly basis, and the brace was removed at seven weeks. The patient was ambulatory at 2 months postoperatively without pain. She could stand on her tiptoes and go running at 3 months postoperatively.

## Discussion

The reliability of lag screw fixation in osteoporotic bone remains questionable. Khazen *et al*. [6] demonstrated in a cadaveric study that lag screw fixation alone failed at a mean load of 251 N, whereas augmentation with suture anchors significantly increased the failure load to 441.6 N. Clinically, Rauer *et al*. [7] reported a case in which two CCS failed on the third postoperative day in a 74-year-old osteoporotic woman. Mitchell *et al*. [8] reported in a multicenter analysis that 28.2% of operatively treated patients experienced fixation failure, with age being the sole predictor of catastrophic failure. These findings suggest that lag screw fixation alone is insufficient in elderly osteoporotic patients.

Badillo *et al*. [5] reported an overall complication rate of 34.1% in their systematic review, with no consensus on the optimal fixation method. A multicenter study of 70 patients further demonstrated that CCS with augmentation was more effective than CCS alone [9]. Ninomiya *et al*. [10] described a Krackow suture secured to a CCS in four patients; however, this construct relies on single-point fixation without footprint restoration.

The technique used in the present case is based on the concept of dual fixation: anatomical reduction and compression of the bony fragment with CCS, combined with footprint restoration using a knotless suture bridge. Beitzel *et al*. [11] demonstrated that the double-row suture bridge construct failed at 434 N, significantly superior to single-row fixation at 212 N. Cox *et al*. [12] reported that knotted suture bridge constructs demonstrated significantly higher ultimate load to failure compared with knotless constructs. However, the posterior heel skin is notably thin, and knot prominence may predispose to wound complications, the most prevalent complication alongside fixation failure [5]. Yoshida *et al*. [13] reported a knotted soft anchor bridge technique for calcaneal avulsion fractures; however, the knotted construct carries the risk of knot prominence in this anatomically vulnerable region. To the best of our knowledge, no reports have combined knotless suture bridges with CCS fixation. By employing a knotless construct, soft-tissue irritation is minimized, while the concurrent CCS fixation compensates for the lower load to failure inherent to knotless configurations.

We present a case of calcaneal tuberosity avulsion fracture treated with a knotless suture bridge (SpeedBridge) and cannulated cancellous screw. This technique may serve as a useful fixation option for calcaneal tuberosity avulsion fractures in elderly women with osteoporosis.

## Data Availability

The datasets used and analyzed during the current study are available from the corresponding author on reasonable request.
